# Experimental gastritis leads to anxiety- and depression-like behaviors in female but not male rats

**DOI:** 10.1186/1744-9081-9-46

**Published:** 2013-12-17

**Authors:** Jia Luo, Tao Wang, Shan Liang, Xu Hu, Wei Li, Feng Jin

**Affiliations:** 1 Key Laboratory of Mental Health, Institute of Psychology, Chinese Academy of Sciences, Beijing, China; 2 University of Chinese Academy of Sciences, Beijing, China

**Keywords:** Gastrointestinal-brain axis, Iodoacetamide-induced gastritis, Anxiety, Depression, Gender difference

## Abstract

Human and animals studies support the idea that there is a gender-related co-morbidity of pain-related and inflammatory gastrointestinal (GI) diseases with psychological disorders. This co-morbidity is the evidence for the existence of GI-brain axis which consists of immune (cytokines), neural (vagus nerve) and neuroendocrine (HPA axis) pathways. Psychological stress causes disturbances in GI physiology, such as altered GI barrier function, changes in motility and secretion, development of visceral hypersensitivity, and dysfunction of inflammatory responses. Whether GI inflammation would exert impact on psychological behavior is not well established. We examined the effect of experimental gastritis on anxiety- and depression-like behaviors in male and female Sprague–Dawley rats, and evaluated potential mechanisms of action. Gastritis was induced by adding 0.1% (w/v) iodoacetamide (IAA) to the sterile drinking water for 7 days. Sucrose preference test assessed the depression-like behavior, open field test and elevated plus maze evaluated the anxiety-like behavior. IAA treatment induced gastric inflammation in rats of either gender. No behavioral abnormality or dysfunction of GI-brain axis was observed in male rats with IAA-induced gastritis. Anxiety- and depression-like behaviors were apparent and the HPA axis was hyperactive in female rats with IAA-induced gastritis. Our results show that gastric inflammation leads to anxiety- and depression-like behaviors in female but not male rats via the neuroendocrine (HPA axis) pathway, suggesting that the GI inflammation can impair normal brain function and induce changes in psychological behavior in a gender-related manner through the GI-to-brain signaling.

## Introduction

The high co-morbidity between gastrointestinal (GI) diseases, including irritable bowel syndrome (IBS), inflammatory bowel disease (IBD) and functional dyspepsia (FD), and psychological symptoms such as depression and anxiety is the evidence for existence of GI-brain axis [1-8]. Many researches on this field have focused on the pathogenic role of psychological stress based on the primary observation that psychological stress induces modifications of motility, secretion, visceral sensitivity, and local inflammatory responses in the GI tract [9,10]. However, in recent years a growing body of studies provides compelling evidences to suggest that not only can the brain affect GI activity, but that the GI activity can also induce changes in brain function [11-13]. There are three potential pathways that have been proposed so far, through which GI signals can be transmitted to and induce changes in brain function: immune (cytokines), neural (vagus nerve) and neuroendocrine (corticosterone/HPA axis) pathways [1,14].

In this study, to examine whether GI inflammation affected psychological behavior, the anxiety- and depression-like behaviors were assessed following induction of gastritis in Sprague–Dawley rats. Gastritis was induced by adding 0.1% (w/v) iodoacetamide (IAA) to the sterile drinking water for 7 days. The activities of the three GI-brain axis pathways through which gastric inflammation may modulate the brain function and behavior were also evaluated. Since these pain-related and inflammatory GI diseases have a considerably higher prevalence in women than in men [15-19], we tested both male and female rats in order to reveal any gender difference in the possible impact of gastritis on psychological behavior. Data provided by ample studies in gonadectomy animals propose sex hormone as an obvious candidate to explain the sexual dimorphism in behaviors [20,21]. Therefore, we also measured the plasma levels of 17ß-estradiol and testosterone in female and male rats respectively.

## Materials and methods A

### Animals

Specific-pathogen-free male and female Sprague–Dawley rats, 8–9 week old, (VITAL RIVER Animal Centre, Beijing, China) were used in the study. All rats were housed individually in wire-mesh cages in animal rooms with controlled temperature at 20 ± 2°C, a relative humidity of 50% − 55%, and a 12:12 h light/ dark cycle. They had free access to standard laboratory rodent chow and fresh sterile drinking water. Four groups of animals were included in the study: control group (C group) and iodoacetamide-treated group (IAA group) of male and female rats. Female rats were housed in another room away from male rats.

### Experiment protocols

After two-week acclimatization (day 1–14), IAA groups were treated with iodoacetamide via drinking water for 7 days (day 15–21). The control groups drank the fresh sterile water throughout the whole experiment. The drinking volumes (ml/g body weight) recorded at experiment days 8 and 15 were used for calculation of the drinking volumes during days 1–14 and 15–21 respectively. Since there is evidence that behavioral test may itself has a gender-related effect on biochemical activity [22], the biochemical markers and psychological behaviors were parallelly assessed in two parts of rats following 7-day IAA treatment. One day after the 7-day IAA treatment period (day 22), each group with 6 rats was sacrificed by decapitation. The trunk blood and gastric tissue were collected, and the brain was removed immediately for further dissection. Simultaneously, a sequence of behavior tests started from day 22 was carried out in each group with 6–8 rats, during which the animals continued to drink either sterile water or water containing IAA. The experimental protocol was approved by the Animal Experiment Ethics Committee of the Institute of Psychology, Chinese Academic of Sciences.

### Induction of gastritis

Iodoacetamide (IAA) was purchased from Sigma Chemical Company, St. Louis, MO, USA. Gastritis was induced by addition of 0.1% (w/v) IAA to drinking water for 7 successive days, based on the model described by Karmeli *et al.* who showed a maximum increase in gastric inflammation and lesion for this period of administration [23]. Since IAA is light sensitive, the light-limiting drink bottles were used, and the IAA-containing drinking water was refreshed every day.

### Behavioral tests

Behavioral tests were conducted in sequential order of sucrose preference test (SPT), open field test (OFT), elevated plus maze (EPM). The SPT examined depression-like behavior, while OFT and EPM were used to evaluate anxiety-like behavior.

### Sucrose preference test (SPT)

This consisted of a 48 hours training session and a 1 hour test session conducted 24 hours after the training session [13,24]. In the training session, singly housed rats were trained for 48 hours to drink sugar water in a cage containing two bottles, one bottle containing 1% sucrose solution and another one containing sterile water. The bottles were switched every 12 hours to prevent possible effects of side preference in drinking behavior. Afterwards, only sterile water was provided for 6 hours. Then food and water were withheld from rats for 18 hours. In the test session, rats got access to two bottles with 1% sucrose solution and water, respectively, for one hour. Sucrose preference was evaluated according to the formula below: Sucrose preference (SP) = [sucrose intake (ml)/(sucrose intake (ml) + water intake (ml))] × 100.

### Open field test (OFT)

The open field apparatus consisted of an arena (100 cm × 100 cm × 40 cm) made of black polypropylene [25]. Rats were individually placed into the corner of the field, and allowed to explore the arena for 5 minutes. Movements were recorded by a video camera mounted above the center of the arena, and analyzed using the ANY-Maze video tracking system (Stoelting CO, USA).

### Elevated plus maze (EPM) test

The EPM apparatus consisted of a center area (10 × 10 cm) with two opposite closed (10 cm wide, 50 cm long, 30 cm high wall at their sides and the far end) and two opposite open arms (10 cm wide, 50 cm long) arranged in the shape of a plus. The device was made of opaque black polypropylene and elevated 50 cm above the floor [26].

Rats were placed individually on the center of the maze facing an open arm and were allowed 5 minutes of free exploration. The movements of the animals during the 5-minute test period were tracked by a video camera positioned above the center of the maze and were analyzed using ANY-Maze (Stoelting CO, USA) video tracking system.

### Determination of myeloperoxidase (MPO) activity in gastric tissue

Full-thickness sample of the gastric corpus (100–120 mg) was excised and the gastric MPO activity was determined according to the previously described spectrophotometric technique [27,28]. MPO is granule-associated enzyme primarily contained in neutrophils, correlates well with the severity of the inflammation and histological lesion, and its measurement has been widely used as a marker of gastrointestinal inflammation. MPO activity was expressed as units per milligram of wet tissue, where one unit of MPO was defined as the quantity of the enzyme able to convert 1 μ mol hydrogen peroxide to water in 1 minute at room temperature.

### Enzyme-linked immunosorbent assay (ELISA) analysis

The trunk blood was collected and centrifuged (1500 × g, 10 min, 4°C). Plasma was taken and immediately stored at −80°C until assayed. Cytokines including interleukin-6 (IL-6), interferon-gama (INF-γ), and tumor necrosis factor-alpha (TNF-α) (IBL, Minneapolis, USA), as well as sex hormones including 17ß-estradiol and testosterone, and corticosterone (Abcam, Cambridge, UK) in plasma were measured using ELISA kits, according to the manufacturer’s instructions.

### RNA isolation and reverse transcription

RNA was isolated from hypothalamus (50–70 mg) using TRNzol reagent according to the manufacturer’s instructions (Tiangen Biotech Co. Ltd., Beijing, China). 4 μl of total RNA from each sample was converted to double-stranded cDNA using TIANScript Reverse Transcription Kit (Tiangen Biotech Co. Ltd., Beijing, China). Subsequently, 2 μl of the resulting cDNA samples were used in the following quantitative real-time PCR (qPCR) for measurement of mRNA expressions of *Gapdh* (housing keep gene), *C-fos*, corticotropin-releasing factor (*Crf*) and glucocorticoid receptor (*Gr*).

### Quantitative real-time polymerase chain reaction (qPCR)

The qPCR reaction was performed in a Real Time PRC system (Applied Biosystems, model 7300) using SYBR® Premix Ex Taq™ (Takara Bio, Japan). The primers used in this study were listed in Table 1. Primers were validated previously to exclude cross reactivity [29-32].

**Table 1:** Primer sequences used for real-time PCR

**Target**	**Forward (5′- 3′)**	**Reverse (5′- 3′)**
GAPDH	ATGACTCTACCCACGGCAAG	TACTCAGCACCAGCATCACC
c-fos	CCGACTCCTTCTCCAGCAT	TCACCGTGGGGATAAAGTTG
CRF	GTTGAATTTCTTGCAACCGGAG	GACTTCTGTTGAGGTTCCCCAG
GR	AGGGGAGGGGGAGCGTAATGG	CCTCTGCTGCTTGGAATCTGC

Relative quantification was analyzed with the 7300 system SDS software, and the mean 2^-△CT^ was calculated for each sample. Results were presented as percentage expression relative to *Gapdh*.

### Statistical analysis

Results were presented as means ± standard error of mean (S.E.M). In all statistical comparisons, two-tailed, *p* < 0.05, were regarded as significant difference. Statistical evaluation of the results was performed on SPSS 17.0 (SPSS Inc., Chicago, IL, USA) with two-way analysis of variance (ANOVA) to identify gender differences, treatment effects and the interaction between these factors. The homogeneity of variance was analyzed with the Levene test. When the significance was reached with the ANOVA, a post-hoc test (Tukey HSD test) was employed.

## Results

### Gastric myeloperoxidase (MPO) activity

**Figure 1:**
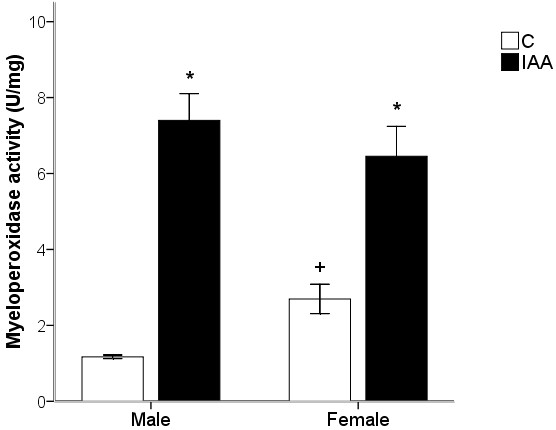
**The IAA treatment induces an increase in gastric MPO activity in rats of either gender.** IAA, iodoacetamide. Values are means ± S.E.M of six rats. ^*^p < 0.05 was significantly different from control group of the same gender. ^+^*p* < 0.05 was significantly different from male control group.

As shown in Figure 1, MPO activity was significantly increased (ANOVA for factor treatment: *F*_(1, 20)_ = 78.59, *p* < 0.001) in IAA-treated rats as compared with control rats. The IAA-induced increase in MPO activity was similar between male and female rats (ANOVA for factor gender: *F*_(1, 20)_ = 0.265, *p* > 0.05), whereas female control rats had a greater (*p* < 0.05) MPO activity than male control rats. The interaction between the factors treatment and gender was significant (*F*_(1, 20)_ = 4.81, *p* < 0.05).

### Behavior test for depression-like behavior

#### Sucrose preference test (SPT)

**Figure 2:**
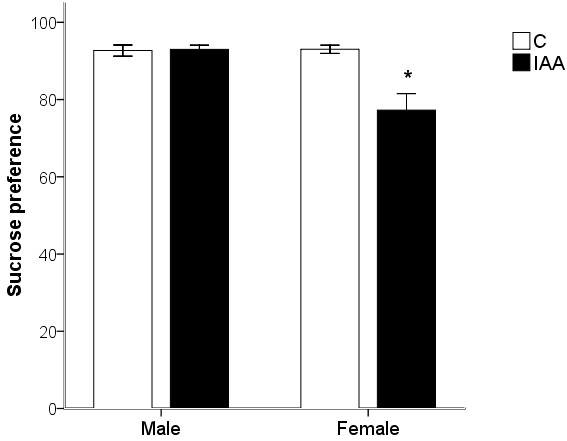
**The female but not male rats with IAA-induced gastritis develop depression-like behavior.** The sucrose preference test was used to measure depression. The sucrose preference correlated negatively with the depression state. IAA, iodoacetamide. Values are means ± S.E.M. ^*^p < 0.05 was significantly different from control group of the same gender. N = 8 rats/male group, N = 6 rats/female group.

In the SPT, the sucrose preference was taken as indices of depression. As shown in Figure 2, the IAA treatment significantly reduced the sucrose preference (ANOVA for factor treatment: *F*_(1, 24)_ = 12.79, *p* < 0.01). Moreover, the interaction between the factors treatment and gender was significant (*F*_(1, 24)_ = 13.80, *p* = 0.001). The sucrose preference was significantly (*p* < 0.05) decreased only in female but not (*p* > 0.05) male IAA-treated rats, while in control rats the sucrose preference did not exhibit any gender difference (*p* > 0.05).

### Behavior test for anxiety-like behavior

#### Open field test (OFT)

In the OFT, the number of entries into, the time spent and the distance traveled in the center zone were taken as indices of anxiety. These parameters were expressed as a percentage of the total entries into, the total time spent and the total distance traveled in the center zone during the 5 min test session.

**Figure 3:**
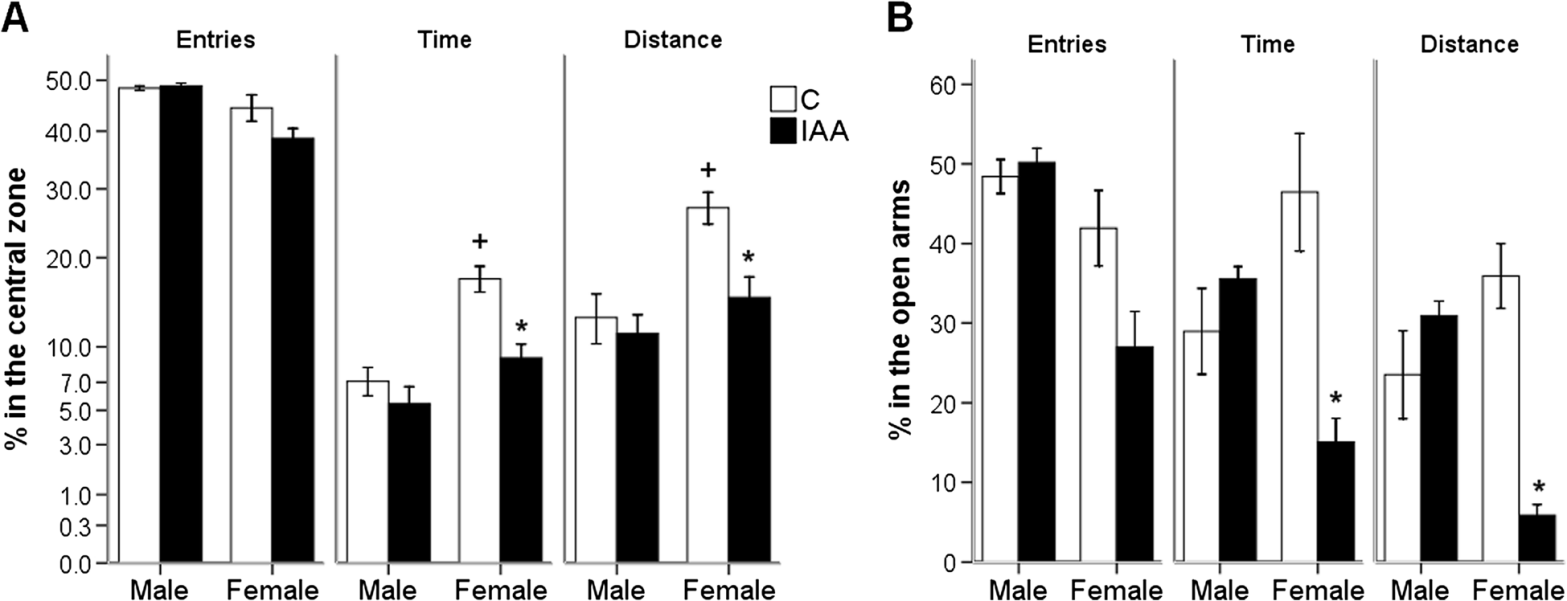
**The female but not male rats with IAA-induced gastritis develop anxiety-like behavior. (A)** Female rats with IAA-induced gastritis spend less time and travel less distance in the central zone of open field test (OFT). **(B)** Female rats with IAA-induced gastritis spend less time and travel less distance in the open arms of elevated plus maze (EPM). The OFT and EPM evaluated the anxiety-like behavior. The percentages of the number of entries into, the time spent and the distance traveled in the center zone of OFT and in the open arm of EPM correlated negatively with the anxiety state. IAA, iodoacetamide. Values are means ± S.E.M. ^*^p < 0.05 was significantly different from control group of the same gender. ^+^*p* < 0.05 was significantly different from male control group. N = 8 rats/male group, N = 6 rats/female group.

The Figure 3A demonstrated that the IAA treatment had no effect on the number of entries into the central zone (ANOVA for factor treatment: *F*_(1, 24)_ = 3.49, *p* > 0.05). The number of entries into the central zone was similar between IAA-treated and control rats of the same gender. The IAA treatment significantly reduced the time spent and distance traveled in the central zone (ANOVA for factor treatment: *F*_(1, 24)_ = 14.91, *p* = 0.001; *F*_(1, 24)_ = 7.98, *p* < 0.01). The interaction between the factors treatment and gender was significant (central zone time: *F*_(1, 24)_ = 6.67, *p* < 0.05; central zone distance: *F*_(1, 24)_ = 4.62, *p* < 0.05). However, the central zone time and distance were decreased (*p* < 0.01; *p* < 0.01) only in female but not male IAA-treated rats, while female control rats spent significantly (*p* < 0.001; *p* < 0.01) more time and distance in the central zone than male control rats.

#### Elevated plus maze (EPM)

In the EPM, the number of entries into, the time spent and the distance traveled in the open arms were taken as indices of anxiety. These parameters were expressed as a percentage of the total entries into, the total time spent and the total distance traveled in any arm during the 5 min test session.

In Figure 3B, the IAA treatment had no effect on the number of entries into the open arms (ANOVA for factor treatment: *F*_(1, 24)_ = 4.18, *p* > 0.05). The number of entries into the open arms was similar between IAA-treated and control rats of the same gender. The IAA treatment significantly reduced the time spent and distance traveled in the open arms (ANOVA for factor treatment: *F*_(1, 24)_ = 6.88, *p* < 0.05; *F*_(1, 24)_ = 8.67, *p* < 0.01). The interaction between the factors treatment and gender was significant (open arm time: *F*_(1, 24)_ = 16.23, *p* < 0.001; open arm distance: *F*_(1, 24)_ = 23.73, *p* < 0.001). However, the open arm time and distance were decreased (*p* < 0.01; *p* < 0.001) only in female but not in male IAA-treated rats, while in control rats the time spent and distance traveled in open arms did not exhibit any gender difference (*p* > 0.05; *p* > 0.05).

### Plasma levels of pro-inflammatory cytokines

Male and female control rats did not differ in plasma concentrations of IL-6 (47.91 ± 2.62 pg/ml *vs* 40.80 ± 2.71 pg/ml, *p* > 0.05), TNF-α (134.10 ± 11.84 pg/ml *vs* 123.88 ± 8.26 pg/ml, *p* > 0.05) and INF-γ (898.68 ± 77.66 pg/ml *vs* 805.09 ± 51.85 pg/ml, *p* > 0.05). Furthermore, the IAA treatment had no effect on plasma levels of IL-6, TNF-α and INF-γ (ANOVA for factor treatment: *F*_(1, 20)_ = 0.27, *p* > 0.05; *F*_(1, 20)_ = 0.29, *p* > 0.05; *F*_(1, 20)_ = 1.66, *p* > 0.05). The concentrations of IL-6, TNF-α and INF-γ did not differ between IAA-treated and control rats of the same gender. Plasma levels of IL-6, TNF-α and INF-γ in male IAA-treated rats were 49.64 ± 3.91 pg/ml, 118.18 ± 8.18 pg/ml and 830.98 ± 44.98 pg/ml, in female IAA-treated rats were 42.41 ± 3.50 pg/ml, 149.95 ± 9.00 pg/ml and 923.51 ± 40.11 pg/ml.

### *C-fos* mRNA expression in hypothalamus

The hypothalamic *C-fos* expressions were similar between male and female control rats (0.54 ± 0.09% *vs* 0.49 ± 0.13%, *p* > 0.05). The IAA treatment had no effect on hypothalamic *C-fos* expression (ANOVA for factor treatment: *F*_(1, 20)_ = 2.723, *p* > 0.05). The interaction between the factors treatment and gender was not significant.

### The activity of hypothalamic–pituitary–adrenal (HPA) axis

#### Corticotropin-releasing factor (Crf) mRNA expression in hypothalamus

**Figure 4:**
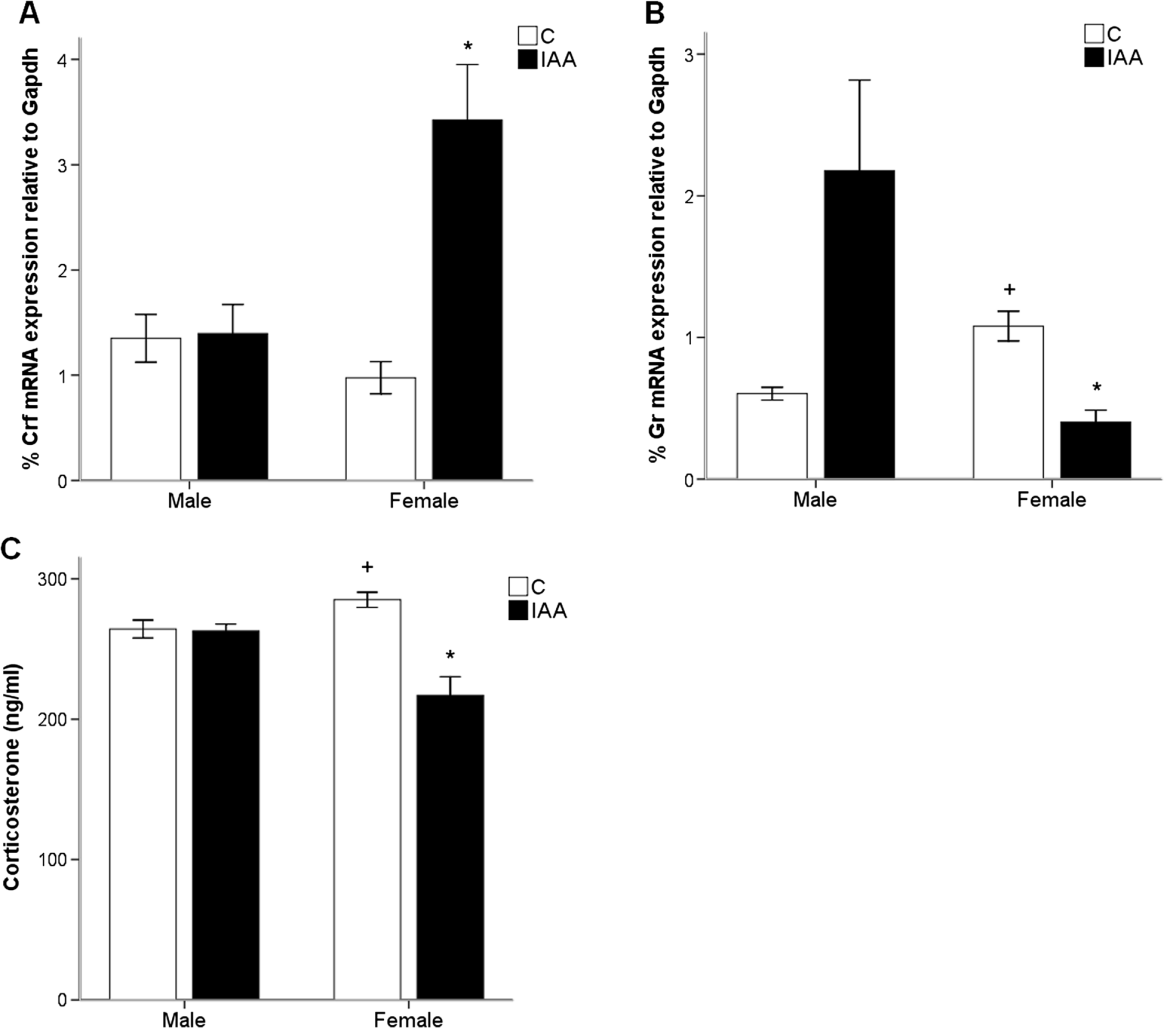
**The IAA treatment induces hyperactivity of HPA axis in female but not male rats. (A) ***Crf* mRNA expression in hypothalamus is increased in female rats with IAA-induced gastritis. **(B) ***Gr* mRNA expression in hypothalamus is reduced in female rats with IAA-induced gastritis. **(C)** Plasma levels of CORT are decreased in female rats with IAA-induced gastritis. The hypothalamic expressions of corticotropin-releasing factor (CRF) and glucocorticoid receptor (GR), and plasma levels of corticosterone (CORT) were used to assess the HPA axis activity. IAA, iodoacetamide. Values are means ± S.E.M of six rats. ^*^p < 0.05 was significantly different from control group of the same gender. ^+^*p* < 0.05 was significantly different from male control group.

As shown in Figure 4A, the IAA treatment significantly increased the hypothalamic *Crf* expression (ANOVA for factor treatment: *F*_(1, 20)_ = 14.56, *p* = 0.001). The interaction between the factors treatment and gender was significant (*F*_(1, 20)_ = 13.52, *p* = 0.001). The hypothalamic *Crf* expression was only increased (*p* = 0.001) in female but not in male IAA-treated rats, while male and female control rats did not differ in hypothalamic *Crf* mRNA expression (1.35 ± 0.23% *vs* 0.97 ± 0.15%, *p* > 0.05).

#### Glucocorticoid receptor (Gr) mRNA expression in hypothalamus

The Figure 4B revealed that the hypothalamic *Gr* mRNA expressions were significantly greater in female than in male control rats (1.08 ± 0.11% *vs* 0.60 ± 0.05%, *p* < 0.01). The interaction between the factors treatment and gender was significant (*F*_(1, 20)_ = 11.85, *p* < 0.01). Furthermore, it was found that IAA treatment induced a drop (*p* < 0.001) in *Gr* expression only in female rats, but not in male rats.

#### Plasma levels of corticosterone (CORT)

In Figure 4C, IAA treatment significantly reduced (ANOVA for factor treatment: *F*_(1, 20)_ = 17.91, *p* < 0.001) plasma CORT levels. The interaction between the factors treatment and gender was significant (*F*_(1, 20)_ = 16.52, *p* = 0.001). The CORT levels were decreased (*p* = 0.001) only in female but not (*p* > 0.05) male IAA-treated rats, whereas female control rats had a higher CORT levels than male control rats (285.12 ± 5.32 ng/ml *vs* 264.38 ± 6.38 ng/ml, *p* < 0.05).

### Plasma levels of sex hormones: 17ß-estradiol and testosterone

**Figure 5:**
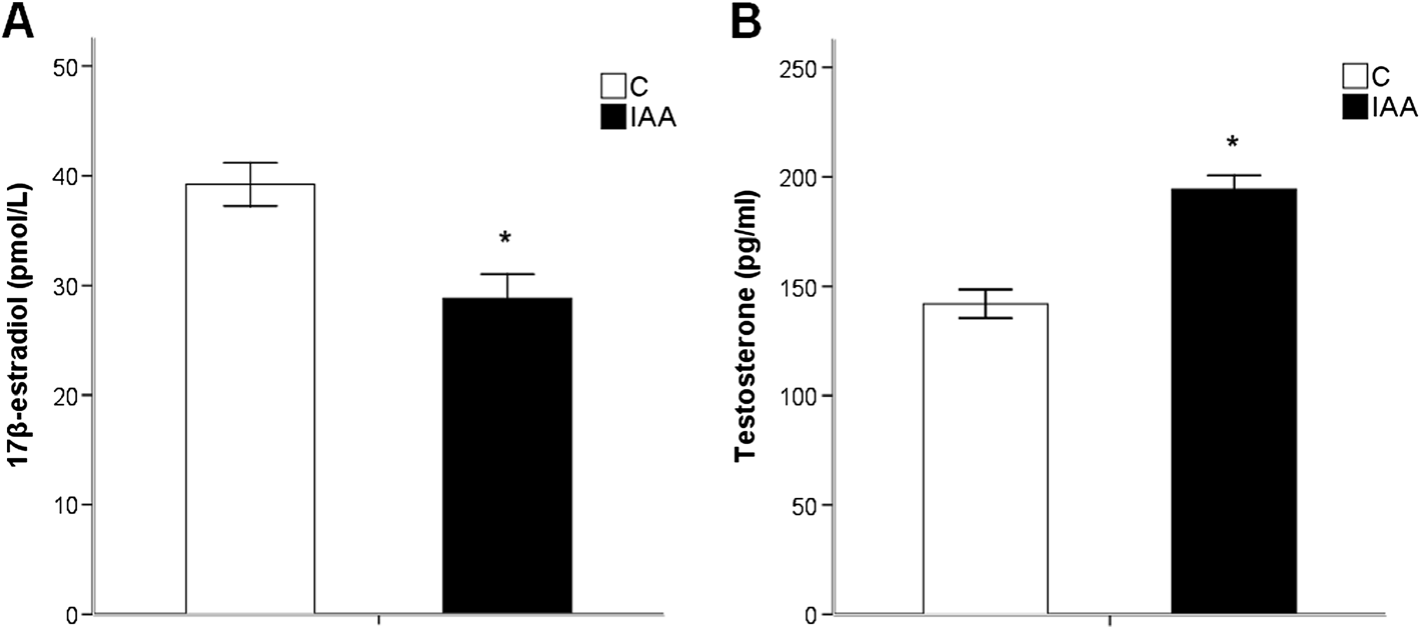
**The IAA treatment causes changes in sex hormones in rats of either gender. (A)** Plasma levels of 17ß-estradiol are decreased in female rats with IAA-induced gastritis. **(B)** Plasma levels of testosterone are increased in male rats with IAA-induced gastritis. IAA, iodoacetamide. Values are means ± S.E.M of six rats. ^*^p < 0.05 was significantly different from control group of the same gender.

IAA treatment induced a reduction (*p* < 0.01) in plasma 17ß-estradiol levels in female rats (Figure 5A). Figure 5B showed that male rats treated with IAA had a higher testosterone level than male controls (194.35 ± 6.37 pg/ml *vs* 141.99 ± 6.56 pg/ml, *p* < 0.001).

### Daily drank water

Rats significantly drank less (ANOVA for factor treatment: *F*_(1, 20)_ = 131.69, *p* < 0.001) IAA water than the normal drinking water. The IAA-induced reduction in drank water was similar between male and female rats (ANOVA for factor gender: *F*_(1, 20)_ = 0.928, *p* > 0.05), while during acclimatization period (day 1–14), the daily drank water was not significantly different (0.160 ± 0.011 ml/g *vs* 0.139 ± 0.011 ml/g, *p* > 0.05) between male and female control rats. As a result, during day 15–21, male and female rats drank similar volume of water-containing IAA (0.0528 ± 0.003 ml/g *vs* 0.0578 ± 0.003 ml/g, *p* > 0.05). The interaction between the factors treatment and gender was not significant (*F*_(1, 20)_ = 2.429, *p* > 0.05).

## Discussion

### Effect of gastric inflammation on psychological behaviors in male and female rats

In view of the growing evidence that pain-related and inflammatory GI diseases are linked to anxiety and depression symptoms, the overall aim of our study was to test a possible relationship between gastric inflammation and psychological behaviors in rats. While it has previously been shown that psychological stressors such as experimentally induced depression- and anxiety-like phenotypes enhance the vulnerability to intestinal inflammation [33-35], we here explore whether GI inflammation has an impact on anxiety- and depression-like behaviors in rats.

The IAA added to drinking water at a concentration of 0.1% for 7 days has been previously shown to elicit murine gastric inflammation. In the present study we observed that the gastric inflammation was successfully induced by IAA treatment in rats of either gender, as assessed by increased MPO activities in IAA-treated rats. A potential drawback of IAA-induced gastritis model is the significant reduction of water intake [36,37]. Nonetheless, previous reported analysis of the circadian activity patterns revealed that IAA reduced drinking, feeding and locomotor activity only during the dark phase to a significant extent, suggesting that the reduction of water intake is not primarily taste-related but, together with the decrease in feeding and locomotor activity, reflects a behavioral consequence of gastritis [22]. The normal appetite for sucrose and water should be reserved in gastritis rats. Moreover, the sucrose preference test has been used extensively to measure anhedonia, a core symptom of depression, in mild stress models of depression [38-40]. Strekalova *et al.*[24] found that anhedonia was associated with analogues of depressive symptoms, such as increased floating during forced swimming and decreased exploration of novelty. Thus, the sucrose preference of IAA-treated rats reflects their depression scores.

The significant interaction between the factors treatment and gender on anxiety- and depression-like behaviors may indicate that the IAA-induced gastritis has a gender-related effect on psychological behaviors. The female rats with IAA-induced gastritis exhibited anxiety- and depression-like behaviors as assessed in SPT, OPT and EPM tests. Nevertheless, IAA treatment failed to affect the psychological behaviors in male rats. Although it could be argued that IAA treatment failed to reduce the time and distance that male rats spent and traveled in the central zone of the open field, because male control rats spent significantly less time and traveled less distance in the central zone than female control rats, this argument is not applicable to the time spent and the distance traveled in the open arms of EPM in which male and female control rats did not significantly differ.

The gender-related behavioral response to experimental gastritis is not an unexpected finding. Firstly, there is clinical evidence for a gender-related co-morbidity of functional dyspepsia with anxiety and depression as we have discussed before [6,8,19]. Secondly, the IAA-induced gastritis in rats has previously been shown to elicit hypersensitivity to both mechanical and chemical noxious stimulation of the stomach, and has been proposed to represent an experimental model of functional dyspepsia [13,37,41].

The gastric inflammation could influence the psychological behavior via the three potential pathways of GI-brain axis including immune, vagal nerve and neuroendocrine pathways. The gender-related increase in anxiety and depression due to gastritis went in parallel with the changes in the three GI-to-brain pathways. The IAA treatment had no effect on pro-inflammatory cytokines levels in plasma. In male rats, a normal behavior is in line with the lack of effect of IAA treatment on pro-inflammatory cytokines. However, the lack of effect of IAA treatment on plasma levels of pro-inflammatory cytokines (IL-6, TNF-α and INF-γ) in female rats may suggest that the gender-related impact of gastric inflammation on psychological behaviors is not mediated by the circulating immune pathway. The vagus nerve is another route for the GI-to-brain signaling. When the vagus nerve is activated, information carried by vagal afferent from gastrointestinal tract is transmitted to the nucleus tractus solitaries (NTS), the site of primary afferent termination of the vagus nerve — before areas involved in the stress response such as the paraventricular nucleus of the hypothalamus [42-44]. It has been shown that stimuli induces expression of c-fos expression in neurons in the NTS, and also in the PVN of hypothalamus [45,46]. Moreover, data provided by studies in vagotomy animals showed that the stress-induced c-fos expression in PVN was reduced by vagotomy [47-49], suggesting the neuronal activity in PVN can reflect the vagus nerves activity.

Expression of c-fos, either *C*-*fos* mRNA or c-Fos protein levels (c-Fos protein-positive neurons), is an indirect marker of neuronal activity because c-fos is often expressed when neurons fire action potentials. Emerging body of literature presents ample evidences that *C-fos* mRNA expression can be used as a tool to assess neuronal activation [50-55]. Moreover, data from previous studies have shown that the *C-fos* mRNA expression was significantly correlated with c-Fos protein expression in neurons of brain regions [56-59]. Therefore, we evaluated the vagus nerve activity by measuring the mRNA expression of *C-fos* in the hypothalamus. There is a nonsignificant interaction between the treatment and gender on hypothalamic *C-fos* expressions. No significant difference in hypothalamic *C-fos* mRNA expressions between female rats with IAA-induced gastritis and female controls may indicate that the vagal nerves do not mediate the gender-related impact of gastric inflammation on psychological behavior in the present study. Nevertheless, it is important to emphasize that the entire hypothalamus may be a relatively extensive size of brain region compared to PVN, and may not be effective in the assessment of vagus afferent activity. Therefore, these results must be interpreted with caution. The observed lack of effect of IAA treatment on hypothalamic c-fos expression seems to be in contrast to the many reports that describe an induction of c-fos expression in a variety of brain areas in response to a wide range of stressors, including restraint, swimming, audiogenic noise and immune challenge [60-63]. However, these stressors that induce c-fos expression are acute stressors. Data from ample studies have suggested that habituation to chronic stress could induce the lack of stress-induced c-fos expression in the PVN of hypothalamus [64-67]. Tan *et al.*[64] have shown that expression of c-fos in the hypothalamic PVN region of the brain was induced and reached a peak at 0.5 hours for *C-fos* mRNA and 4 hours for c-Fos protein, but disappeared at 2 hours for *C-fos* mRNA and 16 hours for c-Fos protein during continuous restraint stress. Moreover, despite the lack of effect of chronic stress on c-fos expression in the PVN, the CRF expression in PVN and plasma CORT levels were found to be markedly changed during chronic stress [64,68,69]. This dissociation between c-fos and CRF expression in PVN during chronic stress is in line with the results of the current study that the 7-day-IAA induced gastritis causes gender-related alterations in hypothalamic mRNA expressions of *Crf* and *Gr* and CORT levels in plasma, but had no effect on hypothalamic C-*fos* mRNA expression.

Either neural excitatory input to the PVN (represented primarily by c-fos expression) or the ability of cells in the PVN to respond to that input (represented primarily by CRF expression) could activate the HPA axis negative feedback [70]. Double-labelling studies have shown that the majority of cells in the PVN that express c-Fos in response to stressful stimuli can also express CRF [71]. The dissociation between hypothalamic C-*fos* and *Crf* mRNA expressions observed in our study may indicate that dysregulation of HPA axis induced by gastritis stress is not a result of increased excitatory neural input to the PVN, but instead depends on some direct effect of gastritis on cells intrinsic to the HPA axis. Similar results were reported in the Sprague–Dawley rat following restraint stress with glucocorticoid pretreatment [70].

The hypothalamic-pituitary-adrenal (HPA) axis plays a prominent role in the neuroendocrine route for the GI-to-brain signaling. The HPA axis activation is a homeostatic mechanism that is triggered by a physical or psychological stress to release corticotropin-releasing factor (CRF) and terminated by the negative feedback action that elevations in circulating CORT inhibit the HPA axis by acting via the glucocorticoid receptor (GR) [72]. The significant interaction between the factors treatment and gender on plasma levels of corticosterone and hypothalamic mRNA expressions of *Gr* and *Crf* may suggest that the IAA-induced gastritis has a gender-related effect on the HPA axis activity. The HPA axis activity was altered in female but not male rats with IAA-induced gastritis, as reflected by decreases in hypothalamic *Gr* mRNA expression and blood CORT levels, and an increase in hypothalamic *Crf* mRNA expression. These alterations are likely to indicate a hyperactivity of HPA axis due to reduced sensitivity to the negative feedback action of CORT. HPA axis hyperactivity is a consistent finding among patients with major depression disorder [73], and anxiety disorders including panic disorder [74], social anxiety disorder [75] and generalized anxiety disorder [76]. Although it could be argued that IAA treatment failed to reduce the hypothalamic *Gr* mRNA expression and plasma CORT levels in male rats, because the hypothalamic *Gr* mRNA expression and plasma CORT levels were significantly less in male than in female control rats, this argument is not applicable to the hypothalamic *Crf* mRNA expression in which male and female control rats did not significantly differ.

Hyperactivity of HPA axis is primarily characterized by an elevation in central CRF expression which also has been demonstrated to play a prominent role in the etiology of anxiety and depression. Intracerebroventricular administration of CRF reduces the open arm exploration in the EPM and also has anxiogenic effect in other anxiety tests [77]. In clinical studies, individuals with depression, anxiety or suicide exhibit more CRF neurons in hypothalamus than normal individuals [78]. Altogether, the gastric inflammation, the hyperactivity of HPA axis, in combination with the anxiety- and depression-like behaviors in female IAA-treated rats suggest that gastric inflammation has a gender-related impact on psychological behaviors via neuroendocrine (HPA axis) pathway.

Rats developed gastric inflammation following 7-day IAA treatment, since the IAA treatment increased the gastric MPO levels in rats of either gender. Furthermore, a significant interaction between the factors treatment and gender in MPO activity may suggest there is a gender difference in sensitivity of IAA-induced gastritis. Although female control rats have a higher MPO levels than male control rats, the IAA treatment still significantly increases the MPO activity in both genders. It may suggest that female rats are more vulnerable to IAA-induced gastritis than male rats. This gender difference in sensitivity of IAA-induced gastritis is consistent with the clinical findings that GI disorder has a considerably higher prevalence in women than in man [15-17]. Therefore, we consider that the gender-related effect of gastritis on anxiety and depression as well as HPA axis activity may be related to the sensitivity to gastritis which was greater in female than in male rats.

### Association between sex hormones and the gender-related anxiety- and depression-like behaviors

Data from animal and clinical studies may also provide some evidences for clarifying the mechanism by which gastric inflammation affects the HPA axis activity and psychological behaviors in a gender-related manner. There is an ample body of literatures that propose the sex hormone as an obvious candidate to explain the behavioral and physiological gender differences [20,21,79].

The sex hormones in female and male rats respectively are 17ß-estradiol and testosterone which are demonstrated to modulate the HPA axis response to stressors. In the rodent, the HPA axis responses to endotoxin and to IL-1 are enhanced by gonadectomy and attenuated by estradiol and testosterone replacement [80]. Data from clinical study have also shown that following a precipitous decline in estradiol levels during pregnancy, postpartum women experience greater HPA axis response to stressors [81]. Therefore, in our study, a decline in estradiol level in female IAA-treated rats may lead to an enhanced HPA axis response to gastric inflammation, while an increase in testosterone level in male IAA-treated rats may reserve a normal HPA axis response to gastric inflammation.

Furthermore, since the HPA axis activity has been shown to regulate psychological behaviors, the alterations in sex hormones may be relevant to the gender-related anxiety- and depression-like behaviors in rats with IAA-induced gastritis. Data from previous studies have provided evidences for an association between decreased sex hormones and increased susceptibility to anxiety and depression. Postpartum depression, as well as premenstrual syndrome, premenstrual dysphoric disorder and menopause depression are all associated with a drop in circulating estrogen [81]. In addition, men with hypogonadism, a condition where the body produces no or low testosterone, suffer increased levels of depression and anxiety, while testosterone replacement therapy has been shown to effectively improve mood [82,83].

It is well known that cyclical female sex hormone variation has a profound effect on behavioral and neurochemical parameters. Although this can be important, the short duration of the estrous cycle in rats coupled with the duration of the study and the fact that female rats housed in the same room can phase into the same estrous cycle stage [84] would most likely result in not having a differential predominance of animals in any one particular phase of the cycle across groups [85], and exclude the possibility of confounding behavioral effects of different phases of the cycle during the same testing sessions [86-89].

## Conclusions

In summary, our results show that the IAA-induced gastric inflammation leads to anxiety- and depression-like behaviors in female but not male rats via the neuroendocrine pathway, suggesting GI inflammation has a gender-related impact on psychological behavior and providing evidence for the existence of GI-to-brain signaling. This gender-related behavioral effect of gastric inflammation may be related to the different sensitivity to gastritis and the changes in sex hormones. Further studies are required to define how these changes, that happen locally in GI, at the molecular level contribute to the regulation of brain function and behavior in a gender-related manner.

## Abbreviations

IAA: Iodoacetamide; GI: Gastrointestinal; IBS: Irritable bowel syndrome; IBD: Inflammatory bowel disease; ELISA: Enzyme-linked immunosorbent assay; qPCR: Quantitative real-time polymerase chain reaction; MPO: Myeloperoxidase; SPT: Sucrose preference test; EPM: Elevated plus maze; OPT: Open field test; HPA: Hypothalamic–pituitary–adrenal; CRF: Corticotropin-releasing factor; GR: Glucocorticoid receptor; CORT: Corticosterone.

## Competing interests

The authors declare that they have no competing interests.

## Author’s contributions

JL analyzed, interpreted the data and drafted the manuscript; JL and FJ contributed to the study concept and design; JL, TW, SL and XH performed the research and took care of the acquisition and analysis of data; JL and WL did critical revision of the manuscript for important intellectual content; TW, XH and FJ provided administrative, technical, or material support; TW, SL, XH and WL contributed essential reagents or tools; FJ supervised the study. All authors read and approved the final manuscript.
